# *Microtetrameres cloacitectus* in Eurasian buzzard (*Buteo buteo*): pathology, phylogenetics, and seasonality

**DOI:** 10.1007/s00436-025-08517-w

**Published:** 2025-07-02

**Authors:** Katarzyna Anna Hołówka, Andrada Negoescu, Marian Taulescu, Angela Monica Ionică, Georgiana Deak, Andrei Daniel Mihalca, Călin Mircea Gherman

**Affiliations:** 1 Department of Parasitology and Parasitic Diseases, University of Agricultural Sciences and Veterinary Medicine of Cluj-Napoca, Calea Mănăștur 3-5, Cluj-Napoca, 400372 Romania; 2 Department of Veterinary Pathology, University of Agricultural Sciences and Veterinary Medicine of Cluj-Napoca, Calea Mănăștur 3-5, Cluj-Napoca, 400372 Romania; 3 Clinical Hospital of Infectious Diseases of Cluj-Napoca, 23 Iuliu Moldovan, Cluj-Napoca-Napoca, 400348 Romania

**Keywords:** *Microtetrameres cloacitectus*, Eurasian buzzard, Proventriculities

## Abstract

*Microtetrameres cloacitectus* is a nematode of the family Tetrameridae Travassos, 1914, parasitising the proventricular glands of diurnal raptors. First described by Oschmarin (1956) from the Eurasian buzzard (*Buteo buteo*) in Primorsky Krai, Russia, it has since been reported in several raptor species. Between 2017 and 2024, 88 deceased Eurasian buzzards were collected from various regions in Romania. For each specimen, GPS coordinates, sex, and age were recorded, and full parasitological necropsies were performed. One individual (over three-year-old male found as roadkill in Tureni, Cluj County, Northwestern Romania) had *Microtetrameres*-like nematodes; a section of its proventriculus was preserved in 10% formalin for histopathological examination. In the remaining positive cases, nematodes were preserved in ethanol for molecular analyses and in 4% formalin for morphological identification. Overall, 25 buzzards were infected with *M. cloacitectus*, resulting in a prevalence of 28.4% (95% CI 20.0–38.6%). A total of 779 specimens (464♀, 315♂) were recovered, with an intensity range of 1–316 parasites (mean = 31.2). Infections showed a seasonal pattern, peaking in summer, and were more frequent in birds over 2 years old. Molecular analysis yielded a novel cox1 sequence and revealed new phylogenetic relationships. Histopathology revealed multifocal glandular distention and inflammatory nodules centred on parasites. This study provides the first comprehensive parasitological, molecular, and histopathological characterisation of *M. cloacitectus* in Romania.

**Supplementary information:**

The online version contains supplementary material available at 10.1007/s00436-025-08517-w.

## Introduction

Family Tetrameridae Travassos, 1914, contains three genera of nematodes: *Geopetitia*, *Microtetrameres*, and *Tetrameres*. All genera parasitise the digestive tract of various species of birds (Dueñas Díaz et al. 2018). *Geopetitia* spp. form cysts in the oesophagus, proventriculus, and gizzard, and each cyst contains multiple females. *Microtetrameres* and *Tetrameres* are located in the proventriculus and females individually occupy gastric glands (Anderson 2000). While *Tetrameres* include mostly waterfowl parasites, species of *Microtetrameres* infect mainly birds of prey (Kinsella and Forrester 2008). There are over 50 known species in the latter genus (Kinsella and Forrester 2008).

The first description of *Microterameres cloacitectus* was provided by Oschmarin (1956) in Eurasian buzzards. Female parasites are reddish, and the body is coiled into a spiral. Sexual dimorphism is strongly marked in this species. Males are easily distinguishable from females, based on the following characteristics: smaller size, with a filiform, cylindrical, and white body (Barus 1966). The female parasites are embedded in proventricular glands. Males are either embedded in proventricular glands with a female or free in the proventriculus lumen (Barus 1966). *M. cloacitectus* was found in several avian hosts, all diurnal raptor species (Table 1).

**Table 1:** Previous reports of *M. cloacitectus* with the host species and the geographical range

Host	Country	Examined/positive (%)	Reference
Eurasian goshawk (*Astur gentilis*)	Germany	13/1 (7.7%)	Honisch and Krone, 2008
Eurasian sparrowhawk (*Accipiter nisus*)	Germany	18/1 (5.6%)	Honisch and Krone, 2008
Eurasian buzzard (*Buteo buteo*)	Czechia	27/2 (7.4%)	Barus, 1966
	Germany	848 (9.5%)	Krone, 2000
	Slovakia	119/8 (6.7%)	Komorová et al., 2017
	Romania	88/25 (28.4%)	Hołówka et al., 2025
Common kestrel (*Falco tinnunculus*)	Czechia	Not mentioned	Sitko and Okulewicz, 2010
	Slovakia	73/2 (2.7%)	Komorová et al., 2017
Western marsh harrier (*Circus aeruginosus*)	Germany	5/1 (20.0%)	Krone, 2000

Despite multiple studies on nematodes of diurnal raptors, considerable gaps in knowledge on *M. cloacitectus* persist. To this point, the life cycle, the parasite’s complete host range, and geographic distribution are not fully known. In addition, the current state of understanding its phylogeny and genetics remains incomplete.

The life cycle of *Microtetrameres* sp. was first described by Cram (1934), who showed that among the tested invertebrate species, grasshoppers (*Melanoplus* spp.) were effective intermediate hosts for *Microtetrameres helix* Cram, 1927. Similar results were obtained by Ellis (1969a, 1969b) and Bethel (1973), in experimental infection of grasshoppers with embryonated eggs of *Microtetrameres centuri* Barus 1966, and *Microtetrameres corax* Schell, 1953*.* A more recent study on the life cycle of *Microtetrameres inermis* (Linstow, 1879; Quentin et al. 1986) proved that *Tylotropidius patagiatus* Karsch, 1893, and *Locusta migratoria* (Linnaeus, 1758) can also serve as effective intermediate hosts, leading to the conclusion that orthopterans represent intermediate hosts for the genus.

There is little data regarding the pathogenic effect of Tetrameridae on their wild bird hosts. Bethel’s (1973) experimentally infected two black-billed magpies (*Pica pica hudsonia*) (Sabine, 1823) with *M. corax* and observed behavioural changes. Infected birds were less active and less aggressive than birds form the control group, which led to decreased food intake and weight loss. In addition, they suffered from multiple ulcerations on the legs and feather loss. Previously performed histopathological examinations of experimentally infected meadowlarks (*Sturnella magna* (Linnaeus, 1758) and *Sturnella neglecta* Audubon, 1844) indicated that *M. centuri* is hematophagous, and the growth of the females may result in atrophy of proventricular glands; additionally, mild inflammatory response surrounding parasites was present (Ellis 1969b). Bethel (1973) found similar results in experimental infections with *M. corax* in black-billed magpies (*P. pica hudsonia*), additionally showing the atrophy of proventricular glands with loss of functionality. Clark et al. (1979) observed dilatation and hyperplasia of duct epithelium followed by atrophy of glandular secretory cells caused by natural infection of *M. nestoris* Black and Rutherford, 1979, in North Island kākā *(Nestor meridionalis septentrionalis*) (Gmelin, JF, 1788). Furthermore, the necrosis and haemorrhage surrounding embedded parasites were also presented. However, up to date, there is no study showing the pathology associated with the infection with *M. cloacitectus*, despite being a common nematode parasitic in diurnal raptors. Moreover, most data on the pathology associated with Tetrameridae originate from experimental infections, with very scarce data on natural infections (Cram 1934, Elis, 1969b, Clark et al. 1979). In addition, no pathological investigations were performed on tetramerids of diurnal raptors.

In this context, the main aim of this study was to investigate the parasitological features, such as a detailed description of microscopic lesions caused by natural infection with *M. cloacitectus*, possible annual fluctuations of infections, and age-specific differences in *M. cloacitectus* infections in Eurasian buzzards from Romania. Additionally, this study provides a new genetic data in order to elucidate the phylogenetic relationship and detailed measurements of the parasites.

## Materials and methods

The carcasses of 88 Eurasian buzzards (*B. buteo*) found dead due to natural causes or as roadkills were collected between 2017 and 2024. For each carcass, the following data were recorded: GPS coordinates, sex based on gonads determined during necropsy (M/F/unknown), and age based on plumage, determined during necropsy (juvenile—less than 1 year old, subadult—between 1 and 2 years old, and adult—older than 2 years old) (Zuberogoitia et al. 2005). The carcasses were stored at − 18 °C until examination, which involved a full parasitological necropsy as previously described in Hołówka et al. (2024, 2025).

When detected, *Microtetrameres*-like nematodes were extracted by applying pressure with a curved tweezer on the gastric mucosa and cleaned in saline solution. Following collection, the parasites were divided equally and preserved in 4% formaldehyde solution for morphological studies, and in absolute ethanol for molecular identification. Place of origin; age; sex; physical parameters such as weight, length of wings, and length of tarsus of the birds; and number of parasites are presented in the supplementary file Supplement 1.

A road-killed male Eurasian buzzard (*Buteo buteo*, > 3 years, male) was collected from Tureni village, Cluj County, Romania (46.6230° N, 23.7033° E). During the necropsy, a 1-cm^2^ section of its proventriculus containing embedded nematodes was excised and preserved in 10% formaldehyde for 24 h. Subsequently, the specimens underwent standard processing procedures, followed by embedding in paraffin wax and sectioning into slices measuring 2–3 µm in thickness using Thermo Scientific™ HM 325 Rotary Microtome. The sections were routinely stained using haematoxylin and eosin (H&E) stain, and images were captured using the Olympus SP 350 digital camera with Stream Basic imaging software (Olympus Corporation, Japan).

Morphological identification of the nematodes was performed based on the detailed description of *M. cloacitectus* given by Barus (1966). Identifications were performed with an Olympus BX61 light microscope (Olympus Corp., Japan), and measurements and pictures were made with dedicated software Cell^F ver. 2.4 (Olympus Corp., Japan).

The DNA was isolated individually from two randomly chosen specimens (one male and one female), using the DNeasy Blood and Tissue Kit (Qiagen, Germany), according to the manufacturers’ protocol. The samples were further processed by means of PCR amplification and bidirectional sequencing of a ∼700 bp fragment of the *Cytochrome c oxidase* subunit 1 (*cox*1) gene, using the NTF/NTR primer pair (Casiraghi et al. 2001). The sequences were compared to other isolates deposited in GenBank by means of Basic Local Alignment Search Tool (BLAST) analysis. The phylogenetic relationships were inferred using MEGA XI software (Kumar et al. 2018). Representative *cox*1 sequences of members of the Habronematoidea superfamily were downloaded from GenBank and aligned together with the two sequences obtained during the present study by MUSCLE alignment. The evolutionary history was inferred by using the maximum likelihood method and Tamura-Nei model (Tamura and Nei 1993), with 1000 bootstrap replicates. A discrete Gamma distribution was used to model evolutionary rate differences among sites (five categories (+ *G*, parameter = 0.3313)).

All data was processed in a tabular database (Microsoft Excel; Microsoft Corp., Redmond, WA, USA). Mean intensity of *M. cloacitectus* infection was calculated by dividing the number of collected parasites by the number of infected birds. Besides that, prevalence (number of hosts of *M. cloacitectus* divided by number of examined Eurasian buzzards multiplied by 100%) and confidence interval (CI95%) were calculated with Epitools Epidemiological Calculators (Sergeant, ESG, 2018).

The study was conducted under the permit numbers 229 from 19/09/2020 and 364 from 06/03/2023 granted by the bioethics committee of USAMV Cluj-Napoca.

## Results

**Fig. 1:**
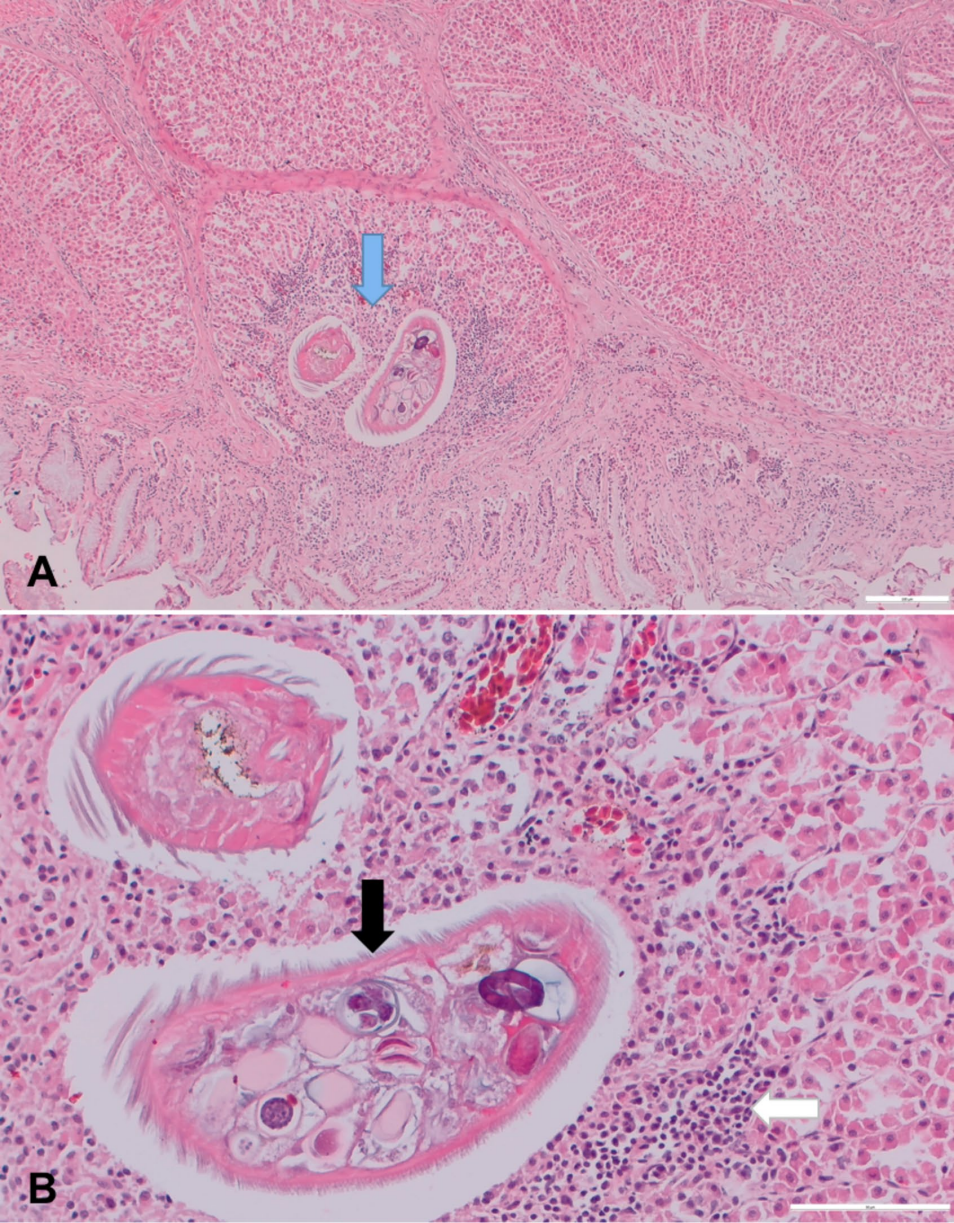
Histological features of proventriculitis caused by *M. cloacitectus* in a Eurasian buzzard *(B. buteo)*: **a** The glandular layer of the proventriculus contains fragments of adult parasites (blue arrow), **b** showing striated cuticle (black arrow) surrounded by inflammatory cells (white arrow). H&E stain. Scale: 100 µm for **a**, 50 µm for **b**

During the necropsies, embedded females were easily detectable in the proventriculus. They appeared as red nodules in the proventricular glands. Histology showed that the glandular layer of the proventriculus was multifocally distended by inflammatory nodules centred on parasitic organisms (Fig. 1a). The inflammatory reaction was moderate and consisted of macrophages, lymphocytes, and plasma cells (Fig. 1b). Mild hyperemia and cell debris were also noted around the parasites.

**Fig. 2:**
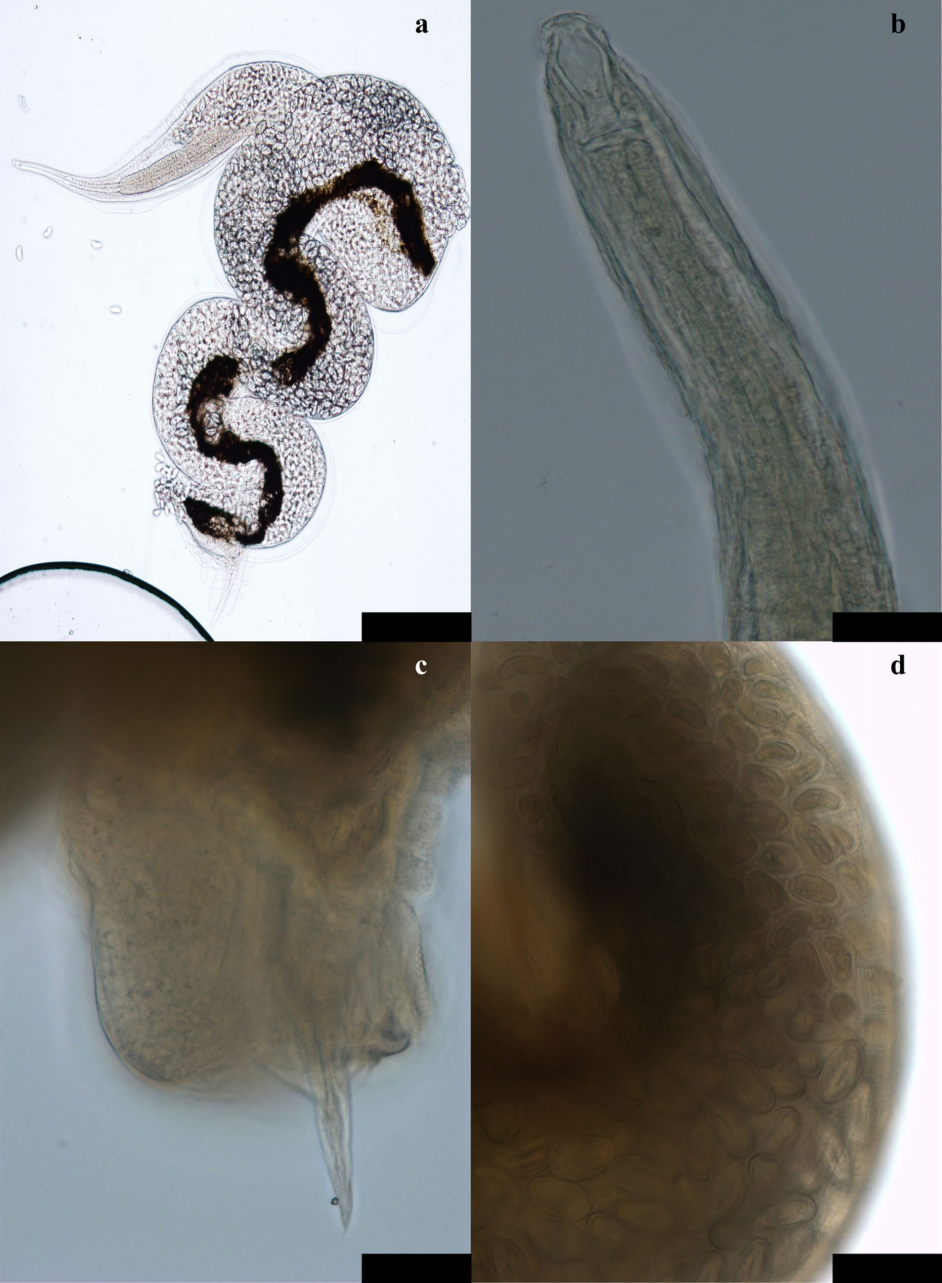
Morphological aspects of *M. cloacitectus* females: **a** general aspect; **b** anterior end of the body; **c** posterior end of the body; **d** eggs containing the 1 st stage larvae. Scale: 500 µm for a, 50 µm for **b**–**d**

**Fig. 3:**
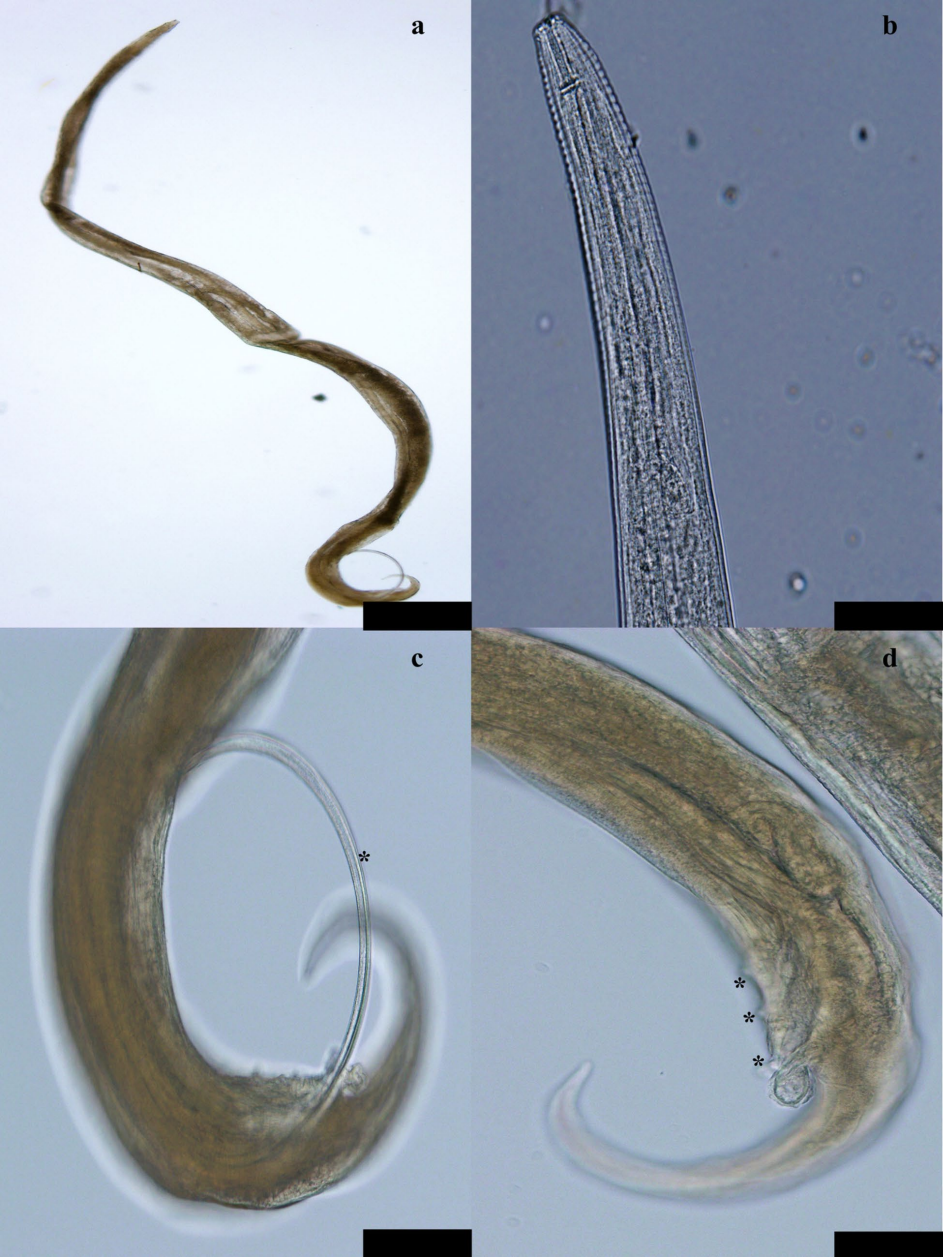
Various aspects of *M. cloacitectus* males: **a** general aspect; **b** anterior end of the body; **c** posterior end of the body with the spicule visible (*); **d** posterior end of the body with the precloacal papillae visible (*). Scale: 500 µm for **a**, 100 µm for **c** and **d**, 50 µm for **b**

Twenty-five out of 88 examined birds were infected with *M. cloacitectus* (*P* = 28.4%, CI 95% = 20.0–38.6%)*.* In total, 779 (464♀, 315♂) specimens of *M. cloacitectus* (Figs. 2 and 3) were collected. Recorded intensity of infection ranged from 1 to 316 parasites, and the mean intensity was equal to 31.2. All the measurements are displayed in Tables 2 and 3.

**Table 2:** Measurements of 12 males *M. cloacitectus* in comparison to Barus (1966) (SE, standard error)

Males	Barus (1966) (mm)	Current study (mm) (SE)
Body length	2.82	2.85 (0.0561)
Maximum width	0.08	0.09 (0.0027)
Buccal capsule depth	0.022	0.024 (0.0007)
Buccal capsule width	0.01	0.015 (0.0004)
Muscular oesophagus length	0.266	0.263 (0.0060)
Muscular oesophagus width	0.011	0.015 (0.0004)
Glandular oesophagus length	0.64	0.63 (0.0186)
Glandular oesophagus width	0.046	0.053 (0.0015)
1 st spicule length	1.07	1.046 (0.0241)
1 st spicule width	0.011	0.019 (0.0006)
2nd spicule length	0.222	0.262 (0.0058)
2nd spicule width	0.008	0.009 (0.0002)
Cuticular sack length	0.012	0.011 (0.0003)
Nervous ganglion position	0.152	0.163 (0.0055)

**Table 3:** Measurements of 24 females *M. cloacitectus* in comparison to Barus (1966) (SE, standard error)

Females	Barus (1966) (mm)	Current study (mm) (SE)
Diameter of body	0.63	0.49 (0.0075)
Maximum body width	0.249	0.22 (0.0054)
Anterior end of body width	0.015	0.019 (0.0003)
Buccal capsule depth	0.026	0.021 (0.0004)
Buccal capsule width	0.009	0.009 (0.0002)
Muscular oesophagus length	0.114	0.266 (0.0026)
Muscular oesophagus width	0.019	0.016 (0.0017)
Glandular oesophagus width	-	0.58 (0.0093)
Length of the eggs	0.042–0.046	0.042 (0.0008)
Width of the eggs	0.026–0.029	0.027 (0.0005)

Statistical analyses showed that in birds that were at least 2 years old, the prevalence of infection was higher. Among all the positive birds, 18 were in that age group (*n* = 18/25, *P* = 72.0%, CI 95% = 54.4–85.7%). In addition, *M. cloacitectus* was found with higher prevalence in birds collected during the summer. During autumn and winter, the number of positive birds was significantly lower. No infected birds were collected in the spring. The number of collected birds per season with prevalence and CI 95% is displayed in Table 4.

**Table 4:** Number positive birds collected per season (*P*-prevalence)

Astronomical season	Examined/infected (*P*)	Mean intensity	Range	Age category (examined/infected)				
				1	2	3	> 3	Unknown
Winter	38/14 (36.8%)	49.7	1–118	6/4	15/5	10/4	7/1	-
Spring	8/0	0	0	2/0	4/0	2/0	0	-
Summer	12/6 (50.0%)	60.7	2–316	4/2	6/4	3/0	0	-
Autumn	19/5 (26.3%)	20.8	1–92	3/1	12/4	3/0	1/0	-
Unknown	10/0	0	0		3/0		1/0	6/0

**Fig. 4:**
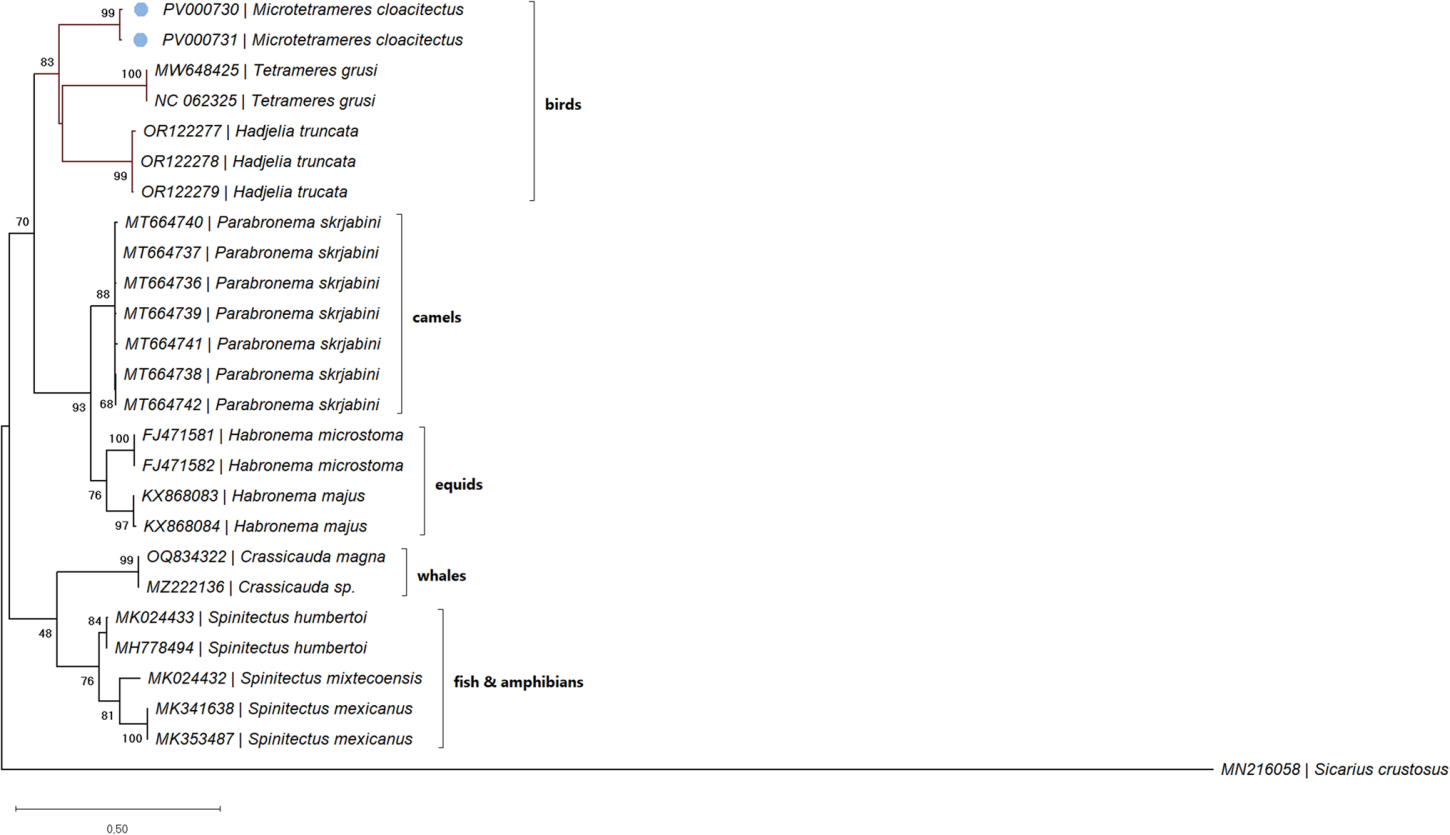
Bootstrap consensus tree inferred from 1000 replicates. The percentage of trees in which the associated taxa clustered together is shown next to the branches (values below 40% not shown). The tree is drawn to scale, with branch lengths measured in the number of substitutions per site. This analysis involved 25 Habronematoidea *cox*1 nucleotide sequences, and one arthropod sequence used as outgroup. There were a total of 438 positions in the final dataset

The highest similarity identified by BLAST analysis (86.31% and 86.76%) was against an unidentified Filarioidea sp. (accession number KP728087). The phylogenetic analysis placed these specimens in a clade comprising parasites of birds (Fig. 4).

## Discussion

Although previous histopathological reports represent studies on different avian hosts and *Microtetrameres* species, there are some similar foundlings. Following studies of Elis (1969b), Bethel (1973), and Clark et al. (1979), the presence of the embedded parasites caused the distension of the glandular layer of the proventriculus. Elis (1969b) reported an inflammatory reaction in the proventriculus caused by *M. centuri* in experimentally infected meadowlarks (*S. magna* and *S. neglecta*), which aligned with the results obtained in this study. Furthermore, histopathological examination confirmed the presence of cell debris and hyperaemia consistent with an inflammatory response, the same as was reported by Clark et al. (1979) in North Island kākā (*N. meridionalis septentrionalis*) which was infected by *M. nestoris*. Contrary to all the authors mentioned above, no gland atrophy was observed.

The histopathological analysis showed no significant evidence of pathogenicity for *M. cloacitectus*. However, the observed inflammatory response suggests that the parasites may negatively affect the host’s health.

Currently, three sequences of *M. cloacitectus* are available in GenBank®. Each sequence is a product of amplification of the ribosomal RNA gene (Friedrich and Krone 2004; Honisch and Krone 2008) (accession numbers: AY702692, AY702693, EU004814). No *cox*1 sequences are available in GenBank, so comparative analysis was not possible.

Recorded prevalence of *M. cloacitectus* in this study was significantly higher in comparison to previous reports. Prior studies from Czechia (Barus 1966), Germany (Krone 2000), and Slovakia (Komorová et al. 2017) reported prevalences of 7.4%, 9.5%, and 6.7%, respectively. Although prevalence in this study was significantly higher, mean intensity was comparable with the results obtained by Krone (2000) and Komorová et al. (2017). Reported mean intensity by those authors was 27.8 and 32.8, respectively. In majority, birds were 2 years old or older, which was probably related to the cumulative effect or to different feeding habits and an increased chance of praying orthopteran intermediate hosts. Šotnár and Obuch (2009) recognised orthopterans, which are suitable intermediate hosts of *M. cloacitectus*, in pellets from Slovakian buzzards. In addition, the season of the collection was a factor that influenced the detection of *M. cloacitectus* in the birds. Obtained results showed a peak of the infection in the summer. This finding could be correlated with the seasonality of orthopterans, as they are generally thermophiles, and their postembryonic development ends in the summer (Finch et al. 2008). However, the exact duration of development within the intermediate host is not known for *M. cloacitectus*. Previous studies indicated that contact with invasive larvae should have occurred at least a month before the collection of the specimens. Bethel (1973) recovered adult parasites from magpies 48 days after infection, while Cram (1934) obtained adult parasites twice from pigeons on the 29th and 35th days following the infections by 3rd-stage larvae. The lack of detection of *M. cloacitectus* in the buzzards collected during spring could be related to the small sample size. This study represents the first one in diurnal raptors which indicates seasonality of infection with *M. cloacitectus*. However, a prior study conducted on Czech populations of grebes and loons (Sitko and Heneberg 2015) demonstrated seasonality of several helminth species occurrence, as well as being connected to the fluctuation of intervertebral hosts activity.

The morphometry of the worms was consistent with Barus (1966). Some small differences related to the females’ body diameter, which in this study was smaller, and muscular oesophagus length which appeared longer in the examined specimens were observed, but these could be related to the fact that the carcass was frozen before examination and the nematodes were preserved in formaldehyde and ethanol for some months (Hass et al. 2024). Nematodes preserved in ethanol appeared darker with the cuticula wrinkled which negatively impacted visibility of internal structures of measured nematodes. While parasites preserved in formaldehyde have well preserved cuticula and appeared lighter in colour in comparison to the nematodes preserved in ethanol. Similar aspects of nematodes based on the medium of preservation were observed by Hass et al. (2024).

## Conclusions

This study is the first report on histopathological findings caused by *M. cloacitectus*. In addition, it is the first comprehensive parasitological study on *M. cloacitectus* indicating the importance of the hosts’ age and showing a potential seasonality of infection. Furthermore, it brings updated knowledge on parasite genetics, providing the first *cox*1 nucleotide sequences which show new phylogenetic relationships. 

## Supplementary information

Below is the link to the electronic supplementary material.

ESM 1 (DOCX 15.3 KB)

## Data Availability

Sequence data that support the findings of this study have been deposited in the GenBank with the accession codes PV000730 and PV000731 https://www.ncbi.nlm.nih.gov/nuccore/PV000731https://www.ncbi.nlm.nih.gov/nuccore/PV000730.
