# Supplementary material for: Microtetrameres cloacitectus in Eurasian buzzard (Buteo buteo): pathology, phylogenetics, and seasonality
Source: Parasitol Res. 2025 Jul 2;124(7):74. doi: 10.1007/s00436-025-08517-w (PMC12222378; doi:10.1007/s00436-025-08517-w)
Supplement: Supplementary file 1 — (DOCX 15.3 KB) [file 436_2025_8517_MOESM1_ESM.docx]

**Supplement 1.** Place of origin**,** date of collection, age, sex, physical parameters of hosts and number of *M. cloacitectus* recorded in Eurasian buzzards from Romania.

| **Place of origin** | **Date of collection** | **Age (years)** | **Sex** | **Weight (g)** | **Number of parasitescollected *M. cloacitectus*** |
| --- | --- | --- | --- | --- | --- |
| Tureni | 09.01.2021 | >3 | M | - | 36 (24♀, 12♂) |
| Petreştii de Jos | 18.08.2021 | 2 | F | 823.3 | 12 (10♀, 2♂) |
| Gilău | 22.09.2021 | 2 | M | 746.8 | Not collected |
| Dumbrăviţa | 31.08.2021 | 2 | M | 539.7 | 3 (1♀, 2♂) |
| Petreştii de Jos | 15.09.2021 | 1 | M | 774.2 | 316 (184♀, 132♂) |
| Turda | 15.09.2021 | 1 | M | 799.2 | 29 (20♂, 9♀) |
| Turda | 07.11.2021 | 2 | F | 844.2 | 1 (1♀, 0♂) |
| Deleni | 06.11.2021 | 2 | F | 805.0 | 9 (8♀, 1♂) |
| Vălişoara | 21.11.2021 | 2 | F | 883.6 | 92 (77♀, 15♂) |
| Ilia | 20.03.2022 | 2 | M | 795.2 | 118 (62♀, 56♂) |
| Agigea | 01.02.2021 | 1 | M | 669.2 | 8 (6♀, 2♂) |
| Mociu | 02.02.2022 | 2 | M | 882.1 | 6 (0♀, 6♂) |
| Deleni | 23.01.2022 | 1 | M | 608.7 | 6 (5♀, 1♂) |
| Tureni | 11.06.2022 | 2 | F | 868.4 | 2 (2♀, 0♂) |
| Iclod | 28.09.2022 | 1 | M | 654.0 | 2 (2♀, 0♂) |
| Apahida | 04.01.2023 | 3 | F | 808.7 | 18 (8 ♀, 10 ♂) |
| Bălțați | 02.02.2023 | 3 | M | 751.3 | 1 (1♀, 0♂) |
| Petreştii de Jos | 03.02.2023 | 3 | M | 895.6 | 5 (5♀, 0♂) |
| Amara | 24.02.2023 | 2 | M | 603.2 | 1 (1♀, 0♂) |
| Amara | 24.02.2023 | 1 | M | 698.3 | 4 (4♀, 0♂) |
| Amara | 24.02.2023 | 2 | M | 637.7 | 97 (43♀, 54♂) |
| Petreştii de Jos | 21.12.2020 | 1 | M | 661.5 | 1 (1♀, 0♂) |
| Sânlazăr | 29.07.2023 | 2 | F | 884.9 | 2 (2♀, 0♂) |
| Cluj-Napoca | 31.01.2024 | 2 | F | 598.9 | 2 (2♀, 0♂) |
| Iași | 05.03.2023 | 3 | M | 769.2 | 18 (7♀, 11♂) |
